# A Novel Teaching System for Industrial Robots

**DOI:** 10.3390/s140406012

**Published:** 2014-03-27

**Authors:** Hsien-I Lin, Yu-Hsiang Lin

**Affiliations:** Graduate Institute of Automation Technology, National Taipei University of Technology, Taipei 106, Taiwan; E-Mail: ken.sofin@gmail.com

**Keywords:** industrial robotic arm, teach pendant, teaching system, Fitts' Law

## Abstract

The most important tool for controlling an industrial robotic arm is a teach pendant, which controls the robotic arm movement in work spaces and accomplishes teaching tasks. A good teaching tool should be easy to operate and can complete teaching tasks rapidly and effortlessly. In this study, a new teaching system is proposed for enabling users to operate robotic arms and accomplish teaching tasks easily. The proposed teaching system consists of the teach pen, optical markers on the pen, a motion capture system, and the pen tip estimation algorithm. With the marker positions captured by the motion capture system, the pose of the teach pen is accurately calculated by the pen tip algorithm and used to control the robot tool frame. In addition, Fitts' Law is adopted to verify the usefulness of this new system, and the results show that the system provides high accuracy, excellent operation performance, and a stable error rate. In addition, the system maintains superior performance, even when users work on platforms with different inclination angles.

## 1. Introduction

Industrial robotic arms are widely used in industrial manufacturing [1]. Operations with robotic arms include packaging and arranging [2], paint spraying [3], welding [4], cutting [5], moving [6], and sanding [7]. Currently, the primary human-machine interface (HMI) for operating a robotic arm is a teach pendant [8–10]. A traditional teach pendant can: (a) control the rotation of any joint in the robotic arms; (b) control the position and pose of an end effector with six degrees of freedom (6-DoF); (c) control the movement speed of the robotic arm; (d) control the input/output (I/O) and communication in the robotic arm, such as air valves, RS-232, and the Ethernet; and (e) create programs enabling the robotic arm to accomplish a series of tasks. Nevertheless, several issues remain regarding the control of robotic arm movement on an established track. For example: (a) accurately controlling an end effector with 6-DoF is extremely difficult and time-consuming because every degree requires an exclusive sequence of button actions; (b) executing robotic arm movements according to scheduled tracks are extremely difficult because of an increased number of interpolation points are necessitated for complicated movements, thereby raising the complexity of operation; (c) improper use of the teach pendant can damage the robotic arm or hurt the operators; and (d) the teach pendant cannot be operated intuitively, which is not efficient. Because of these problems, robotic arms are not sufficiently user-friendly and can only be competently directed by experienced operators. Consequently, numerous factory owners are not willing to incorporate robotic arms into their manufacturing processes.

Compared to previously developed teach pendants, HMI and human-computer interfaces (HCI) technologies have become sufficiently advanced to enable people to operate robotic arms easily. For example, researchers have attached sensors to human arms or bodies to control robotic arms through body movement. In [11] accelerometers attached were to both human arms to control a robotic arm; the right arm controlled the X, Y and Z directions in the Cartesian system, and the left arm controlled the orientation. The authors of [12] attached electromyography (EMG) sensors to a right human arm, which controlled the movement and rotation of a robotic arm, and the opening and closing of the attached claw. In [13] and [14] the various joint movements captured by motion sensors were recorded and the results applied in controlling robotic arms. Although these studies enabled users to operate robotic arms intuitively, the methods were unable to calculate the optimization accuracy for the endpoint position of the robotic arms, rendering these methods incapable of positioning and posing the endpoint of the robotic arms accurately. Consequently, these robotic arms were only used for tasks with simple movements.

In addition, numerous researchers have adopted joysticks and haptic devices to teach and control the robots. In [7] a joystick was used and a force/torque sensor attached to the endpoint of a robotic arm; fuzzy compliance control was applied to direct the robotic arm strength for sanding. In [15] the authors used a Wii remote and headset to control a robotic arm. The Wii remote recognized human actions and controlled the 3D movement and rotation of the robotic arm, and the headset recognized human voice commands. In addition, a force-torque sensor was attached to the end point of the robotic arm to control strength. In [16–18] the Phantom Omni 6-DoF force-feedback joystick was used to control robotic arms, which enabled the operators to feel the feedback from the robotic arms and robot environment. Such a system enabled users to control robotic arms accurately and conduct delicate tasks in a small workspace, such as surgery [16,17]. However, in this system, the work space is limited by the tools (*i.e*., joysticks and haptic devices); therefore, this system cannot be employed when large work spaces are required. Although scale conversions can be performed, accuracy is lost, which is unfavorable because numerous factory tasks are operated in large spaces [1].

Because the traditional teaching system is complicated, this study proposes a new system for teaching movement paths. The proposed system provides users with a fast, intuitive, and accurate teaching method that is applicable to any robotic arm and any work space, and that aids users with accomplishing path teaching quickly. This new system is composed of a teaching tool and a motion capture system. The teaching tool is shaped like a pen for ergonomics purposes and is called a teach pen. The teach pen is a pointing device, by which an operator can accurately record the position and pose (position and orientation) of a target. Through the motion capture system, an optical marker attached to the teach pen collects information related to position and pose. When the operator moves the teach pen to a desired position and pose, the path of movement is planned and recorded easily. In addition, the teach pen incorporates three lock buttons and one non-lock button for facilitating commands and tasks, such as saving movement paths and opening or closing the claw. By inputting corresponding commands using these buttons, the entire movement plan for the robotic arm can be completed.

To verify whether the proposed teaching system is ergonomics and provides superior performance, Fitts' Law [19,20] and the ISO 9241-9 [21] were adopted to analyze and compare traditional systems with the proposed system. Fitts' Law and the ISO 9241-9 are frequently employed to evaluate the performance of the HMIs or HCIs; for example, performance for a mouse [22], stylus [19], joystick [22], and pointing or drag-drop interfaces, (e.g., touchscreen [23]). When evaluating a HMI according to Fitts' Law, the primary method is to conduct an experiment in which the mean time of movement is recorded. By examining the relationship between the mean time of movement and index of difficulty, the performance of the HMI can be determined. ISO 9241-9 is formulated on the basis of Fitts' Law and has been adopted to collect experimental data, such as the mean time of movement and error rate, for statistical calculation. The statistical results are usually suitable for calculating the time of movement in different work environments.

## 2. Material and Methods

**Figure 1.**
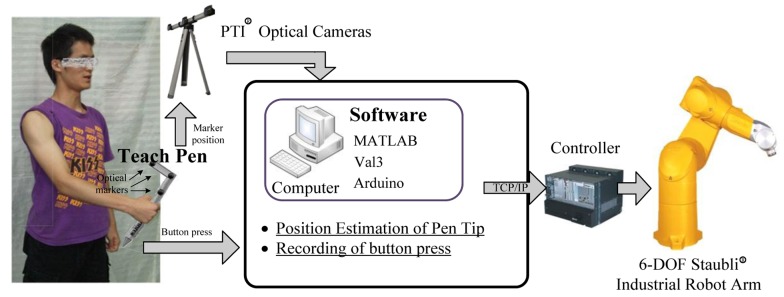
System overview.

The new teaching system proposed in this study can be applied to different types of robotic arms and facilitate the teaching tasks and movement path planning. The main purpose of this study is to identify the tip coordinates teach pen pose. of the teach pen and the position and angle of the pen body. With these coordinates the pen pose, a robotic arm can move accurately as directed by the tip of the teach pen. To elucidate, the new teaching system is divided into three parts, specifically, hardware, software, and computation (Figure 1), as listed below.

### 2.1. Hardware

#### 2.1.1. Teach Pen

**Figure 2.**
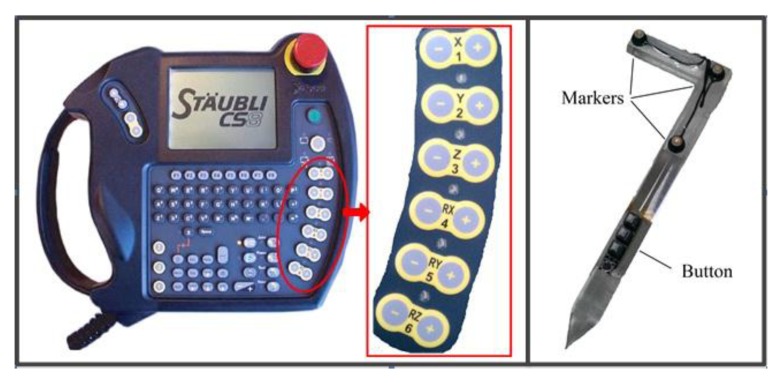
(**a**) The traditional teach pendant; (**b**) the proposed teach pen.

A comparison between the traditional teach pendant and teach pen is shown in Figure 2. The teach pendant (Figure 2a) controls the 6-DoF of the robotic-arm endpoint by twelve + and – buttons, which is not intuitive or convenient. By contrast, the teach pen (Figure 2b) resembles the number “7” and has three active markers, three lock buttons, and one non-lock button. The three markers collect data related to the pen pose and coordinates, and calculate the pen tip coordinates and the pose of the rigid arm body using a proposed algorithm. Concurrently, the signals from the buttons are processed using the Arduino MCU development kit, which controls the opening and closing of the claw.

#### 2.1.2. The Motion Capture System

VZ4000, an optical 3D motion tracker developed by PhoeniX Technologies Inc. (New Taipei, Taiwan) was employed to process the coordinates collected by the three markers on the pen and the two active markers on the operator arm to a measurement accuracy of 0.6 mm and a sample frequency of 2 KHz. The data collected by the optical markers on the pen and the operator arm become zero or inaccurate when the relative viewing angle between the capture system and the marked point is near the operation borderline. Therefore, marked data must be pretreated and filtered to obtain accurate results.

#### 2.1.3. The Industrial Robotic Arm

The robotic arm employed in this study is the STAUBLI TX40 (Pfäffikon, Switzerland), which has 6-DoF, 0.02 mm repeatability, 515 mm jib length, and a maximum speed of 8.2 m/s at the endpoint.

### 2.2. Software

The motion capture system collects the coordinate data saved by the active markers in a specific space, and the computer calculates the coordinates for the pen tip and the pose of the pen based on the data obtained from the active markers. The results are then transmitted via Ethernet to the robotic arm controller for movement control. Because a high sampling rate increases the calculation load and lowers efficiency, 100 Hz was set as the data collection rate for collecting data from the markers.

### 2.3. Computation

#### 2.3.1. Calculating the Position and Pose of the Robotic-Arm Endpoint

**Figure 3.**
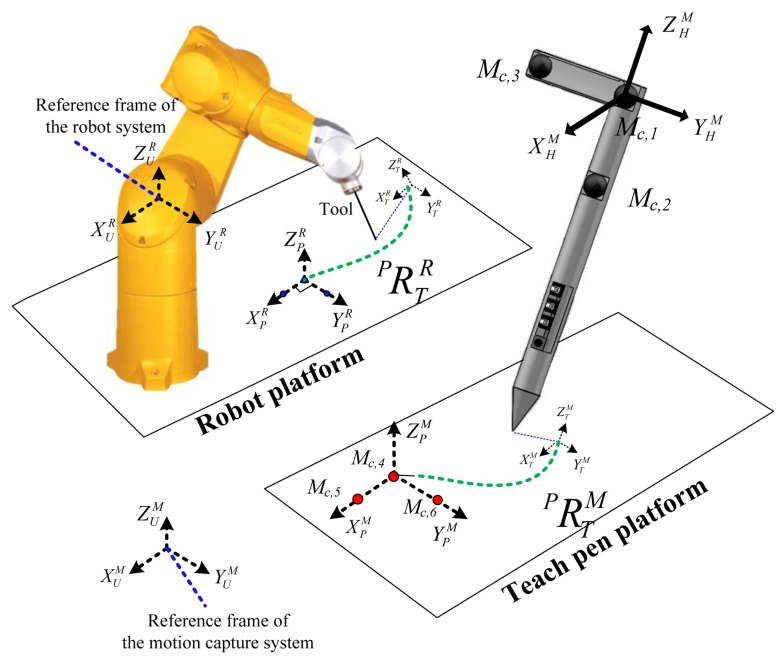
Relationship between the coordinate system of the teach pen and that of the robotic arm.

The teach pen has three markers, namely, *M_C_*_,1_, *M_C_*_,2_, and *M_C_*_,3_ (Figure 3), on which the calculations of the pose and position of the rigid body are based. First, a coordinate system for the teach pen, ( $X_{H}^{M}Y_{H}^{M}Z_{H}^{M}$), must be selected, where $Z_{H}^{M}$ is the direction of *M_C_*_,2_ *M_C_*_,1_, $Y_{H}^{M}$ is the direction of *M_C_*_,3_ *M_C_*_,1_, and $X_{H}^{M}$ is the direction of the cross-product of $Y_{H}^{M}$ and $Z_{H}^{M}$.

The correction method for the teach pen platform is as follows: place three mutually-perpendicular markers on the platform of the pen (M_C,4_, M_C,5_, and M_C,6_; the red spots in Figure 3). The coordinates collected by these markers (*i.e*., the reference markers of the motion capture system; ${\text{X}}_{\text{U}}^{\text{M}}{\text{Y}}_{\text{U}}^{\text{M}}{\text{Z}}_{\text{U}}^{\text{M}}$) can then be used to establish a reference coordinate system for the teach pen platform ( ${\text{X}}_{\text{P}}^{\text{M}}{\text{Y}}_{\text{P}}^{\text{M}}{\text{Z}}_{\text{P}}^{\text{M}}$). Similarly, select three mutually-perpendicular points on the robotic arm platform (the blue spots in Figure 3); the teach pendant is then employed to move the endpoint of the robotic arm to these coordinates. Eventually, the coordinates which are opposite to those of the robotic arm ( ${\text{X}}_{\text{U}}^{\text{R}}{\text{Y}}_{\text{U}}^{\text{R}}{\text{Z}}_{\text{U}}^{\text{R}}$) are obtained, and a reference coordinate system for the robotic arm platform ( ${\text{X}}_{\text{P}}^{\text{R}}{\text{Y}}_{\text{P}}^{\text{R}}{\text{Z}}_{\text{P}}^{\text{R}}$) can thus be created. ( ${\text{X}}_{\text{T}}^{\text{R}}{\text{Y}}_{\text{T}}^{\text{R}}{\text{Z}}_{\text{T}}^{\text{R}}$) represents the coordinate system for the tip endpoints of the robotic arm corresponding to the reference coordinate ( ${\text{X}}_{\text{U}}^{\text{R}}{\text{Y}}_{\text{U}}^{\text{R}}{\text{Z}}_{\text{U}}^{\text{R}}$).

The correction calibration method for the teach pen platform is as follows: Place three mutually-perpendicular markers on the platform of the pen (M_C,4_, M_C,5_, and M_C,6_; the red spots in Figure 3). The coordinates collected by these markers (*i.e*., the reference markers of the motion capture system; ${\text{X}}_{\text{U}}^{\text{M}}{\text{Y}}_{\text{U}}^{\text{M}}{\text{Z}}_{\text{U}}^{\text{M}}$) can then be used to establish a reference coordinate system for the teach pen platform ( ${\text{X}}_{\text{P}}^{\text{M}}{\text{Y}}_{\text{P}}^{\text{M}}{\text{Z}}_{\text{P}}^{\text{M}}$). Similarly, select three mutually-perpendicular points on the robotic arm platform (the blue spots in Figure 3); the teach pendant is then employed to move the endpoint of the robotic arm to these coordinates. Eventually, the coordinates which are opposite to those of the robotic arm ( ${\text{X}}_{\text{U}}^{\text{R}}{\text{Y}}_{\text{U}}^{\text{R}}{\text{Z}}_{\text{U}}^{\text{R}}$) are obtained, and a reference coordinate system for the robotic arm platform ( ${\text{X}}_{\text{P}}^{\text{R}}{\text{Y}}_{\text{P}}^{\text{R}}{\text{Z}}_{\text{P}}^{\text{R}}$) can thus be created. ( ${\text{X}}_{\text{T}}^{\text{R}}{\text{Y}}_{\text{T}}^{\text{R}}{\text{Z}}_{\text{T}}^{\text{R}}$) represents the coordinate system for the tip endpoints of the robotic arm corresponding to the reference coordinate ( ${\text{X}}_{\text{U}}^{\text{R}}{\text{Y}}_{\text{U}}^{\text{R}}{\text{Z}}_{\text{U}}^{\text{R}}$).

#### 2.3.2. Pen Tip Estimation

A virtual marker was adopted for pen tip estimation. The virtual marker is attached to the tip of the teach pen for obtaining the coordinates from the tip. Set *M_C_*_,4_ on the teach pen platform as the origin, and move the tip to this point. Using Equation (3), convert coordinates ( $X_{H}^{M}Y_{H}^{M}Z_{H}^{M}$) of the teach pen into the transformation matrix RPHM for the coordinates ( $X_{P}^{M}Y_{P}^{M}Z_{P}^{M}$) of the teach pen platform. This transformation matrix is then transferred back to the coordinates ( $X_{H}^{M}Y_{H}^{M}Z_{H}^{M}$) of the teach pen for the estimation of the coordinates of the pen tip as $P_{T}^{M}$, which corresponds to the coordinates ( $X_{H}^{M}Y_{H}^{M}Z_{H}^{M}$) on the teach pen. The matrix of ( $X_{H}^{M}Y_{H}^{M}Z_{H}^{M}$) is:

(1) $${\text{T}}_{\text{H}}^{\text{M}}={\left[\begin{array}{cccc} {\text{X}}_{\text{H}}^{\text{M}} & {\text{Y}}_{\text{H}}^{\text{M}} & {\text{Z}}_{\text{H}}^{\text{M}} & {\text{M}}_{\text{C},1}\\ 0 & 0 & 0 & 1 \end{array}\right]}_{4\times 4}$$

The matrix of ( $X_{P}^{M}Y_{P}^{M}Z_{P}^{M}$) is:

(2) $${\text{T}}_{\text{P}}^{\text{M}}={\left[\begin{array}{cccc} {\text{X}}_{\text{P}}^{\text{M}} & {\text{Y}}_{\text{P}}^{\text{M}} & {\text{Z}}_{\text{P}}^{\text{M}} & {\text{M}}_{\text{C},4}\\ 0 & 0 & 0 & 1 \end{array}\right]}_{4\times 4}$$

The RPHM is:

(3) RPMH=(THM)−1×THM

The ( $P_{T}^{M}$) is:

(4) [PT,xMPT,yMPT,zM1]=(RPMH)−1×[0001]

The pose (orientation and position) of the robot ( ${\text{X}}_{\text{T}}^{\text{R}}{\text{Y}}_{\text{T}}^{\text{R}}{\text{Z}}_{\text{T}}^{\text{R}}$), the coordinate system for the tip endpoints of the robotic arm corresponding to the robot reference coordinate ( ${\text{X}}_{\text{U}}^{\text{R}}{\text{Y}}_{\text{U}}^{\text{R}}{\text{Z}}_{\text{U}}^{\text{R}}$), is calculated as:

(5) [Orientation3×3Position3×10001]4×4=RPHM×[100PT,xM010PT,yM001PT,zM0001]

When the teach pen moves to any point on the platform, transformation matrices RPHM can be obtained constantly on the basis of ( ${\text{X}}_{\text{H}}^{\text{M}}{\text{Y}}_{\text{H}}^{\text{M}}{\text{Z}}_{\text{H}}^{\text{M}}$) and ( ${\text{X}}_{\text{P}}^{\text{M}}{\text{Y}}_{\text{P}}^{\text{M}}{\text{Z}}_{\text{P}}^{\text{M}}$), and the new data can be integrated with the ${\text{P}}_{\text{T}}^{\text{M}}$.

By calculating RPTM, which corresponds to transformation matrix between ( ${\text{X}}_{\text{T}}^{\text{M}}{\text{Y}}_{\text{T}}^{\text{M}}{\text{Z}}_{\text{T}}^{\text{M}}$) and ( ${\text{X}}_{\text{P}}^{\text{M}}{\text{Y}}_{\text{P}}^{\text{M}}{\text{Z}}_{\text{P}}^{\text{M}}$) (the transformation matrix represents the pose of the teach pen with respect to the the coordinate of the teach pen platform), and further incorporating this matrix into the robotic arm platform, the coordinate system of the robotic-arm end point can be obtained; that is, (XTRYTRZTR)=RPTM⋅(XPRYPRZPR). This method can be adopted to position and pose the robotic arm as desired.

### 2.4. Fitts-Law-Based Quantitative Evaluation

Fitts adopted an experiment to examine the index of performance (IP), which was changed to throughput (TP) in ISO 9241-9. TP is obtained on the basis of the index of difficulty (ID) and the movement time (MT). According to the Shannon formulation [24], ID refers to the relationship between the distance of movement and the target width:

(6) $$\text{ID}=\log_{2}\left(\frac{\text{D}}{\text{W}}+1\right)$$

*ID* is measured by bits. *D* represents the distance between the start point and the target, and *W* represents the target width. After several experiments, *W* was revised to *W_e_*; thus, the effective index of difficulty (*IDe*) is:

(7) $${\text{ID}}_{\text{e}}=\log_{2}\left(\frac{\text{D}}{{\text{W}}_{\text{e}}}+1\right)$$

In this equation:

(8) $${\text{W}}_{\text{e}}=4.133\sigma$$

(9) $${\text{W}}_{\text{e}}=\{\begin{array}{cc} \text{W}\times \frac{2.066}{\text{Z}(1-\text{Err}/2)} & \text{if Err}>0.0049\%\\ \text{W}\times 0.5089 & \text{otherwise} \end{array}$$

*W_e_* represents the effective target width and can be obtained through Equations (8) or (9). If the discrete endpoint data of the operator pointing actions are collected, the standard deviation (σ) of these actions can be obtained, and *W_e_* can be obtained by Equation (8). If the discrete end-point data cannot be collected, the *W_e_* can be obtained through Equation (9). In this equation, *Err* refers to the error rate in the experiment (the definition of error is provided in the following section) and *z* refers to the z-distribution.

Equation (7) proves that a greater *D* and a smaller error tolerance for *W_e_*, increases *ID*. By contrast, a lower *D* and a greater the error tolerance for *W_e_*, reduces *ID*. If ISO 9241-9 was used to describe the relationship between the *ID* and the *MT*, the following can be expressed:

(10) $$\text{MT}=\text{a}+\text{b}?{\text{ID}}_{\text{e}}$$

In this equation, intercept a and slope b are coefficients obtained by processing the experimental data using linear regression. Basing on *IDe* and *MT*, *TP* can be:

(11) $$\text{TP}=\frac{{{\text{ID}}_{\text{e}}}_{\text{average}}}{{\text{MT}}_{\text{average}}}$$

*TP* is measured by bits/sec (bits per second, bps) and is the performance indicator for the experimental tools. The teach pendant and the teach pen are compared by their *TPs*.

### 2.5. Design of Experiment

#### 2.5.1. Pen Tip Estimation

Nine men (mean age: 23) were selected for this experiment; all of which were right-handed and familiar with the use of a pen. All participants underwent the teach pen experiment, and six underwent the teach pendant experiment.

#### 2.5.2. Apparatus

The equipment employed in this experiment included the new teaching system proposed in this study and the traditional teach pendant. Regarding this new system, an Intel Pentium Dual-Core E6500 2.93 GHz system with 2 GB RAM and running Windows XP SP3 was adopted for this experiment. Regarding the teach pendant, STAUBLI TX40 and a CS8C controller were employed. In this experiment, FlexiForce force sensors, the Arduino MCU development kit, a 180 × 90 × 75 teaching platform, a 37 × 37 × 47 cm chair, and a 100 × 100.5 × 82 cm robotic arm platform were used.

#### 2.5.3. Environment

**Figure 4.**
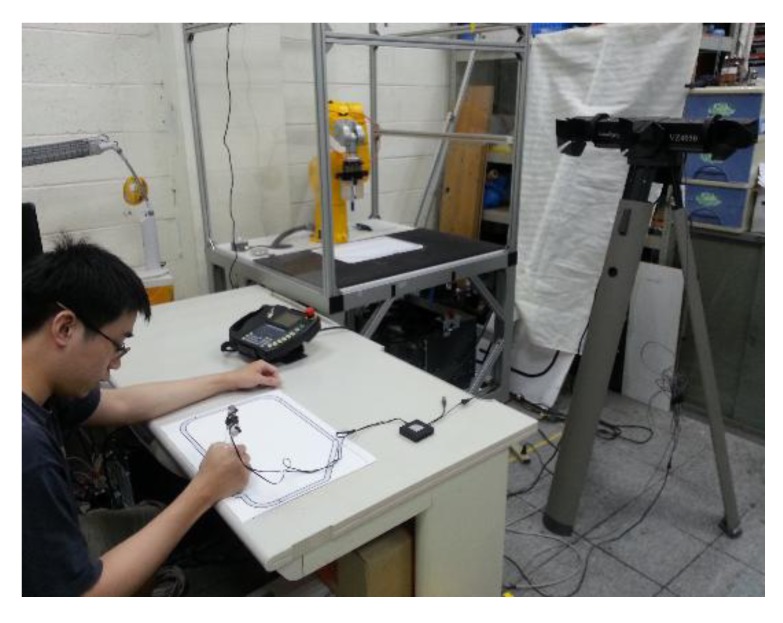
Environment setting of the proposed teaching system.

A subject performed the experiments in a bright and clear environment. There were two working desks: one was for the subject and the other was for the robot. The motion capture system was set up in front of the subject working desk (see Figure 4). The subject held the proposed teach pen where the optical markers were captured by the motion capture system. Since the optical markers were active, their acquired positions were slightly affected by the environmental lighting condition. When the subject moved the teach pen, the robot tool frame was controlled to move to the desired pose.

#### 2.5.4. Procedure

**Figure 5.**
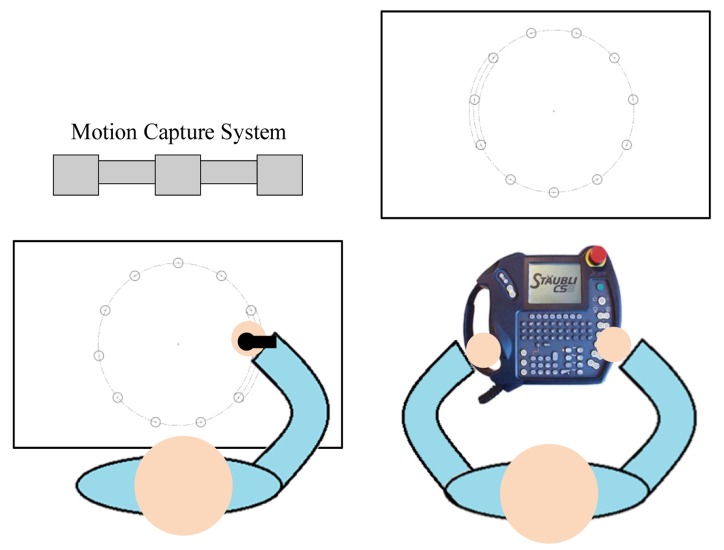
Participants sat or stood at the experiment platform, using (a) the teach pen or (b) the teach pendant.

**Figure 6.**
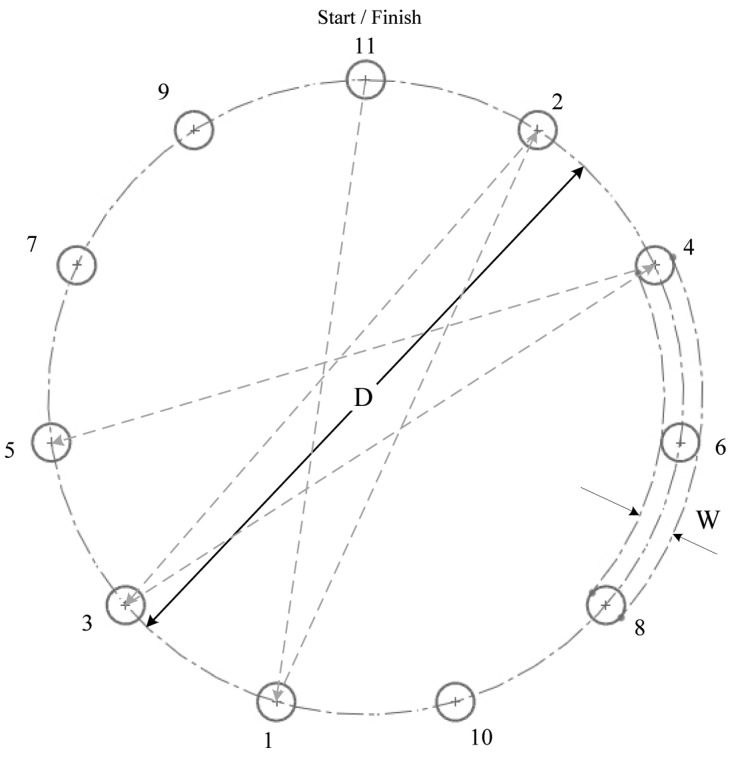
Missions: Participants pointed from Target 1 to Target 11.

Participants sat or stood near the experiment platform (Figure 5) and used the teach pen or the teach pendant as requested. Before experimentation, explanations and practice were provided to the participants, allowing them to familiarize themselves with the tools. In the 10-min practice session, they performed various experiment tasks, such moving the robotic arm to various distances and at various widths, and selecting established targets in a clockwise direction. Tasks were designed according to ISO 9241-9. Figure 6 shows the participants using the teach pen or the traditional teach pendant to move the robotic arm from Targets 1 to 11 (Start/Finish). Prior to timing, the participants were asked to use the teach pen or the teach pendant to move the end point of the robotic arm to the starting point, that is, Target 11 (Start/Finish). The timing began as soon as the robotic arm left the starting point; the arm was required to point from Target 1 to Target 11 in a clockwise direction. The experiment ended as soon as the robotic arm was returned to Target 11 (Start/Finish). After practicing, participants operated the teach pen or the teach pendant to perform the actions shown in Figure 6. They were required to perform the same task five times, during which the MT, error rate, ID, distance, width, and the angle of inclination were recorded and analyzed.

Before operating the teach pendant, participants were provided with training, and were instructed to complete an outcome evaluation. The procedures were as follows: (1) participants were guided through the basic-level operations, such as moving the robotic-arm endpoint in three dimensions and rotating; (2) the participants underwent unassisted practice to familiarize themselves with the experiment tool; (3) an evaluation was conducted to determine the practice outcome of the participants; the difficulty parameters were set at an ID of 3.75, a distance of 150 mm, and a width of 12 mm. Participants who complete the evolution in two minutes with an error rate lower than 10% (based on the test results of most participants) were prepared for actual experimentation.

In the experiment, participants performed tasks in increments of one ID per 15 min session over intervals of 1 h to prevent fatigue from prolonged operating times, which may consequently result in biased data collection. Operation difficulty was increased by one ID following each interval.

Regarding the experiment platform, the timer in the Arduino MCU development kit and FlexiForce force sensors were adopted to measure the time. A force sensor was placed under Target 11 (Start/Finish), which activated the timer when the endpoint of the robotic arm left Target 11 and halted when the endpoint returned to Target 11 when the operation cycle was complete. The times of each operation cycle was collected to determine the MT of each participant.

#### 2.5.5. Experimental Parameters

- Tools: A teach pendant and a teach pen
- Number of targets: 11
- Target width: 6 and 12 mm
- Distance: 150 and 250 mm
- Angle of inclination: 0°, 15°, and −15°

**Figure 7.**
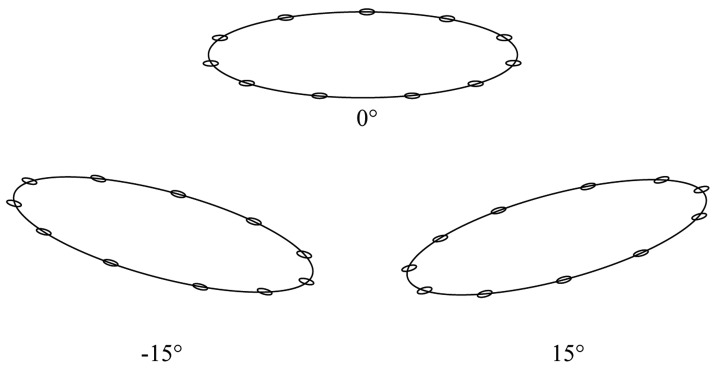
Inclination angles of the experiment platform (0°, 15°, and –15°).

The experimental tools included a teach pendant and a teach pen. Because eleven targets were established, two target widths (6 and 12 mm), two movement distances (150 and 250 mm), four IDs (ID = 2.75, 4.70, 4.45, and 5.41), and three inclination angles (0°, 15°, and −15°; Figure 7) were employed. Overall, 900 pieces of data were gathered (teach pen: 9 participants × 4 IDs × 3 inclinations × 5 repeated missions; teach pendant: 6 participants × 4 IDs × 3 inclinations × 5 repeated missions).

#### 2.5.6. Experimental Considerations

The experiment platform was the work platform for the robotic arm. Because the teach pen controlled the robotic arm directly, the participants used the teach pen to complete the tasks. For safety, the experiment platform was placed away from the work platform of the robotic arm. The participants performed according to the following instructions:

- Adjust to the most comfortable position.
- Point the targets and accomplish the tasks as fast and precisely as possible.
- Practice for 5 min prior to formal experimentation.
- Attempt the assigned tasks five times based on the given instructions.
- Initially, the endpoint of the robotic arm remains on Target 11 (Start/Finish). The timer is activated once the robotic arm endpoint begins to move. Point from Target 1 to Target 11 in a clockwise direction as instructed. The experiment is completed once the endpoint returns to Target 11.
- During the experiment, failure to point at the correct target, same target selected twice or more, or operational failures (e.g., the robotic arm hits the experiment platform and causes the machine to shutdown), an error is recorded and the experiment continues.
- On completion, the duration and the number of errors are recorded.
- An interval of 3 min is provided on each successful completion of a given task.

#### 2.5.7. Experiment Considerations

(1) The paired-samples *t* test was adopted to calculate the mean time, error rate, and the TPs that were recorded in the teach pen experiments and in the teach pendant experiments to determine whether a significant difference exists between the two tools under identical ID and inclination angle conditions.
(2) Repeated-measures ANOVA was adopted to analyze the mean time, error rate, and the TPs recorded in the teach pen experiments to determine whether the mean time, error rate, and the TPs changed significantly under varying ID and inclination angle conditions, and the interaction of both.

## 3. Results and Discussion

### 3.1. Bias and Variance

When operating an industrial robotic arm, accuracy is crucial, and robotic-arm teaching tools also require accuracy. In this article, bias and variance analyses were conducted to signify the degree of accuracy. Bias refers to the difference between the value obtained in one measurement and the known value. If the difference between numerous obtained values and the known value is small, bias is small, and vice versa. Bias is indicated by absolute error, *E*:

(12) $$\text{E}=\bar{\text{x}}-{\text{x}}_{\text{t}}$$

In this equation, *x̅* represents the known value and *x_t_* is the value of an individual measurement.

Variance refers to the degree of concentration among multiple values; a high degree of concentration signifies low variance, and vice versa. Variance is represented by the standard deviation, *σ*:

(13) $$\sigma =\sqrt{\frac{\sum_{\text{i}=1}^{\text{n}}{\left({\text{x}}_{\text{i}}-\bar{\text{x}}\right)}^{2}}{\text{n}-1}}$$

In this equation, *x_i_* refers to the value obtained in the *i*th measurement, *n* refers to the number of measurements, and *x̅* refers to the mean of all values. In the experiment, an operator was instructed to use the teach pen to point at the nine targets on the platform and repeated 100 times to obtain the bias and variance of the teach pen operations. Bias and variance were calculated by Equations (12) and (13). Regarding bias, the mean absolute error was obtained by averaging the values obtained in the 100 measurements and the known value. The results are presented in Table 1.

The table shows the mean absolute error and the standard deviation on the x-axis and the y-axis. Because the experiment was conducted on a surface, the highly accurate z-axis was not presented. On the x-axis, the maximum mean absolute error was only 1.6 mm and the maximum standard deviation *σ* was 1.2 mm. On the y-axis, the maximum mean absolute error was 0.6 mm and the maximum standard deviation *σ* was 0.2 mm. In this study, the mean absolute error indicates that a 1.6 mm error occurred when the teach pen platform was established; this rate could be reduced by rectifying and reestablishing the coordinate system. The source of the error was caused by light and other environmental factors which affected the active markers.

### 3.2. Fitts-Law-Based Quantitative Evaluation

**Figure 8.**
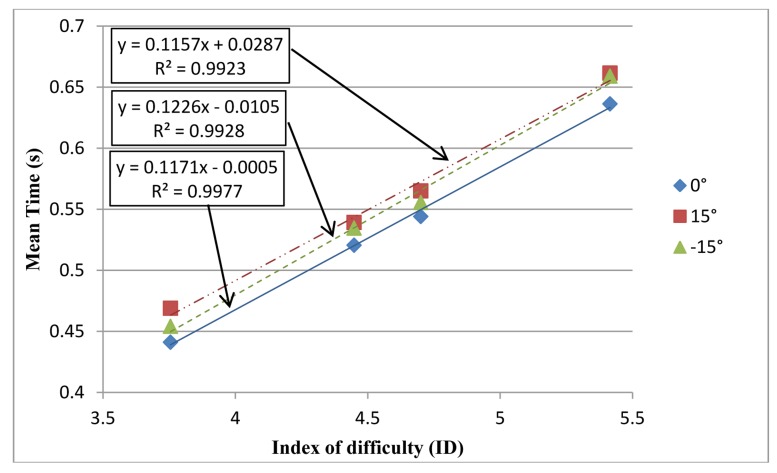
Mean time of the teach pen operation at different IDs and inclination angles.

Table 2 shows the MT equation, correlation, and TP of operating the two tools at three inclination angles. The table indicates that the TP of the teach pen operation was superior to that of the teach pendant operation at all angles. Figure 8 shows the mean time of the teach pen operation at different IDs and on different inclination angles. The MT equation is obtained by linear regression.

#### 3.2.1. Mean Time

Table 3 shows the mean time of operating the two tools at three different inclination angles; the mean time for teach pen operation was considerably less than that for operating the teach pendant. Furthermore, a paired-samples *t* test analysis was conducted and the results are shown in Table 4. The table shows that a significant difference existed between the mean time required by the teach pen operation and that required by the teach pendant operation under identical ID and the inclination angle conditions (*p* < 0.001). The table shows that the mean time required for the teach pen operation was considerably less than that required for the teach pendant, thereby indicating that the teach pen required less time to accomplish missions.

Repeated-measures ANOVA was conducted to compare the mean time required by teach pen operation under varying ID and inclination angle conditions, and the interaction of both. The results are presented in Table 5, and show that a significant difference existed (*F* = 371.802, *p* < 0.05). Furthermore, Figure 8 indicates that a higher ID (the more difficult a task) mandates longer mean time.

A significant difference existed in the mean time required by the teach pen operation among differing inclination angles (*F* = 7.739, *p* < 0.05). Figure 8 shows that the lowest mean time occurred when the inclination angle was 0°, and the sequence was 0° < –15° < 15°. No significant difference existed in the interaction between ID and inclination angle (*p* > 0.05).

#### 3.2.2. Error Rate

Table 6 shows the error rate for operating the two tools at three different inclination angles. Following the paired-samples t test, the results were obtained, as shown in Table 7. The table shows that a significant difference (*p* < 0.001) existed in the error rate for operating the two tools at angle: ID = 5.42 angle = 0°. Further analysis indicated that the large difference (teach pen: 9.09%, teach pendant: 4.55%) was caused by the high ID and the limited time that the participants were allowed for each task.

When ID = 5.42 and angle = 0°, the mean time necessitated by the teach pen operation was substantially less than that by the teach pendant operation. No significant difference existed in the error rate for the other two angles, indicating that no large difference existed.

Table 8 shows a comparison of the error rate for operating the two tools under varying ID and inclination angle conditions, and the interaction of both. A significant difference existed in the error rate for the teach pen operation at varying IDs (*F* = 10.007, *p* < 0.05).

**Figure 9.**
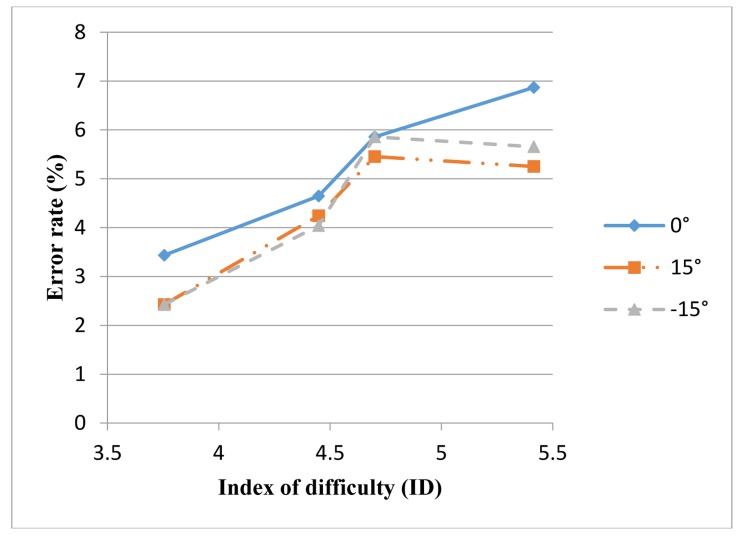
Error rate for the teach pen operation at different IDs and inclination angles.

Figure 9 indicates that a higher ID (a more difficult mission) increased the error rate. No significant difference existed in the error rate for the teach pen operation at varying inclination angles (*F* = 2.852, *p* > 0.05), indicating that the error rate did not vary significantly when the teach pen was operated under varying angles. Finally, no significant difference existed in the error rate for the teach pen operation in the interaction of the ID and the inclination angle (*p* > 0.05).

#### 3.2.3. Throughput

**Figure 10.**
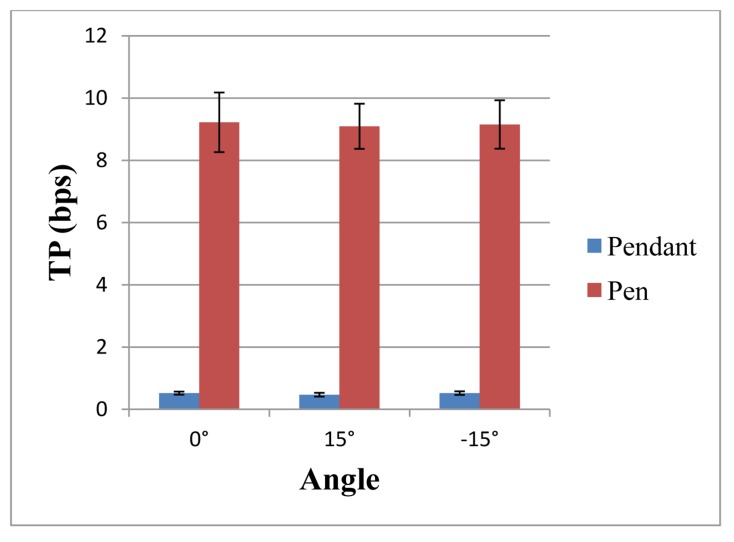
Bar chart of the TP for operating the two tools at three inclination angles.

Figure 10 shows the TP of operating the two tools at three inclination angles; the TP of the teach pen operation was considerably greater than that of the teach pendant operation. Following paired-samples *t* test, the results are presented in Table 9. The table shows that at the same inclination angle, a significant difference existed in the TP between the teach pen operation and the teach pendant operation (*p* < 0.001). Further analysis reveals that the TP of the teach pen operation was considerably greater than that of the teach pendant operation, thereby indicating that the teach pen has superior performance.

Table 10 contains a comparison of the TP of the teach pen operation at different inclination angles; the table shows that no significant difference existed in the TP of the teach pen operation at different angles (*F* = 0.380, *p* > 0.05). Therefore, the differences in angles did not affect the TP of the teach pen operation, thereby indicating that the teach pen had similar performance under varying angles; that is, the superior performance of the teach pen was not affected by the angles of inclination.

## 4. Conclusions

The new ergonomics teaching system proposed in this study demonstrated high accuracy, was easy and intuitive for professional execution, and was less prone to errors. As suggested in Table 1, bias and variance analyses revealed that an average error of only 1.3 mm occurred in the new teaching system. In addition, the operation time and performance of the new teaching system and those of the teach pendant were compared according to Fitts' Law, and the results showed that the new teaching system was superior to the teach pendant for both time and performance. In addition, the error rate for the new teaching system was similar to that for the teach pendant, thereby indicating that accuracy was not negatively affected by short learning time. Moreover, superior performance was demonstrated at all inclination angles.

## Figures and Tables

**Table 1.** Bias and variance in the experimental results.

**Device**	**Target Position**	**Mean Absolute Error**		**Standard Deviation σ**	

		**x (mm)**	**y (mm)**	**x (mm)**	**y (mm)**
Teach Pen	(5,10)	1.07	0.17	1.06	0.17
	(5,20)	1.25	0.19	1.16	0.17
	(5,30)	1.15	0.59	1.09	0.18
	(15,10)	1.67	0.27	1.07	0.22
	(15,20)	1.32	0.20	1.07	0.19
	(15,30)	1.42	0.60	1.02	0.15
	(25,10)	1.48	0.22	1.19	0.22
	(25,20)	1.02	0.41	1.00	0.19
	(25,30)	1.02	0.46	0.84	0.15

Mean	-	1.27	0.34	-	-

**Table 2.** MT equation, correlation, and TP of the two tools at three inclination angles.

**Device**	**Angle (°)**	**MT**	**(R^2^)**	**TP (bps)**
Teach Pen	0	0.0005 + 0.1171 × ID	0.9977	9.22
	15	0.0287 + 0.1157 × ID	0.9923	9.09
	–15	0.0105 + 0.1226 × ID	0.9928	9.15

Teach Pendant	0	1.9285 + 1.7599 × ID	0.9279	0.52
	15	2.5341 + 1.7406 × ID	0.8356	0.47
	–15	0.7965 + 1.9153 × ID	0.7359	0.52

**Table 3.** Mean time, standard deviation, and standard error operating the two tools at three different inclination angles.

**Device**	**Angle (°)**	**Mean Time (s)**	**Std. Deviation**	**Std. Error**
Teach Pen	0	0.54	0.056	0.008
	15	0.56	0.050	0.007
	−15	0.55	0.051	0.008

Teach Pendant	0	9.99	0.848	0.155
	15	10.51	0.862	0.157
	−15	9.57	0.875	0.160

**Table 4.** Paired-samples *t* test table for the mean time operating the two tools at the same ID and inclination angle.

**Source**	**Compare Group**	**N**	**Mean (s)**	**Std. Deviation**	***t* value**
ID = 3.75 Angle = 0°	Teach Pen	30	0.45	0.07	–74.42 ***
	Teach Pendant	30	8.61	0.62	
ID = 3.75 Angle = 15°	Teach Pen	30	0.47	0.05	–68.11 ***
	Teach Pendant	30	8.95	0.70	
ID = 3.75 Angle = –15°	Teach Pen	30	0.45	0.05	–42.69 ***
	Teach Pendant	30	7.96	0.97	
ID = 4.70 Angle = 0°	Teach Pen	30	0.53	0.06	–28.94 ***
	Teach Pendant	30	9.71	1.75	
ID = 4.70 Angle = 15°	Teach Pen	30	0.56	0.06	–50.02 ***
	Teach Pendant	30	10.09	1.05	
ID = 4.70 Angle = –15°	Teach Pen	30	0.56	0.06	–40.55 ***
	Teach Pendant	30	8.75	1.12	
ID = 4.45 Angle = 0°	Teach Pen	30	0.51	0.06	–71.99 ***
	Teach Pendant	30	10.00	0.72	
ID = 4.45 Angle = 15°	Teach Pen	30	0.55	0.07	–61.93 ***
	Teach Pendant	30	10.94	0.93	
ID = 4.45 Angle = –15°	Teach Pen	30	0.54	0.07	–58.51 ***
	Teach Pendant	30	10.15	0.92	
ID = 5.42 Angle = 0°	Teach Pen	30	0.62	0.10	–46.75 ***
	Teach Pendant	30	11.64	1.30	
ID = 5.42 Angle = 15°	Teach Pen	30	.66	0.08	–44.46 ***
	Teach Pendant	30	12.04	1.43	
ID =5.42 Angle = –15°	Teach Pen	30	0.67	0.08	–55.97 ***
	Teach Pendant	30	11.42	1.09	

*** *p* < 0.001.

**Table 5.** ANOVA for the mean time of the teach pen operation.

**Source**	**SS**	**DF**	**MS**	**F**	***p***
ID	2.554	3	0.851	371.802	0.000
Angle	0.034	2	0.017	7.739	0.001

**Table 6.** Error rate, standard deviation, and standard error for operating the two tools at three inclination angles.

**Device**	**Angle (°)**	**Error Rate (%)**	**Std. Deviation**	**Std. Error**
Teach Pen	0	5.51	3.861	0.576
	15	4.14	3.762	0.561
	–15	4.50	3.409	0.508
Teach Pendant	0	3.41	2.784	0.508
	15	5.76	4.456	0.814
	–15	5.38	3.505	0.640

**Table 7.** Paired-samples *t* test table for the error rate for operating the two tools at the same ID and inclination angle.

**Source**	**Compare Group**	**N**	**Mean (%)**	**Std. Deviation**	***t* value**
ID = 3.75 Angle = 0°	Teach Pen	30	3.33	5.06	1.44
	Teach Pendant	30	1.52	4.19	
ID = 3.75 Angle = 15°	Teach Pen	30	3.33	5.59	−1.14
	Teach Pendant	30	5.15	6.62	
ID = 3.75 Angle = –15°	Teach Pen	30	3.03	4.97	−0.77
	Teach Pendant	30	3.94	4.58	
ID = 4.70 Angle = 0°	Teach Pen	30	6.06	6.89	1.19
	Teach Pendant	30	3.94	6.17	
ID = 4.70 Angle = 15°	Teach Pen	30	5.45	6.13	−0.42
	Teach Pendant	30	6.06	6.01	
ID = 4.70 Angle = –15°	Teach Pen	30	5.76	6.08	−0.47
	Teach Pendant	30	6.36	6.82	
ID = 4.45 Angle = 0°	Teach Pen	30	3.64	5.65	0.00
	Teach Pendant	30	3.64	5.65	
ID = 4.45 Angle = 15°	Teach Pen	30	4.24	4.61	0.49
	Teach Pendant	30	3.64	5.65	
ID = 4.45 Angle = –15°	Teach Pen	30	4.85	5.19	1.37
	Teach Pendant	30	2.73	5.92	
ID = 5.42 Angle = 0°	Teach Pen	30	9.09	7.55	2.92 ***
	Teach Pendant	30	4.55	6.20	
ID = 5.42 Angle = 15°	Teach Pen	30	6.06	7.29	−1.07
	Teach Pendant	30	8.18	7.68	
ID = 5.42 Angle = –15°	Teach Pen	30	5.45	6.58	−1.90
	Teach Pendant	30	8.48	7.14	

*** *p* < 0.001.

**Table 8.** ANOVA results for the error rate of the teach pen operation.

**Source**	**SS**	**DF**	**MS**	**F**	***p***
ID	844.812	3	281.604	10.007	0.000
Angle	180.288	2	90.144	2.852	0.063

**Table 9.** Paired-samples *t* test table for the TP for operating the two tools.

**Factor**	**Compare Group**	**N**	**Mean (bps)**	**Std. Deviation**	***t* value**
Angle = 0°	Teach Pen	30	9.40	0.98	49.42 ***
	Teach Pendant	30	0.52	0.05	
Angle = 15°	Teach Pen	30	8.95	0.71	65.58 ***
	Teach Pendant	30	0.47	0.06	
Angle = −15°	Teach Pen	30	9.01	0.88	53.66 ***
	Teach Pendant	30	0.52	0.06	

*** *p* < 0.001.

**Table 10.** ANOVA TP results for teach pen operation.

Source	SS	DF	MS	F	*p*
Angle	0.366	2	0.183	0.380	0.685
